# Phase III study of cisplatin with pemtrexed or vinorelbine plus concurrent late course accelerated hyperfractionated radiotherapy in patients with unresectable stage III non-small cell lung cancer

**DOI:** 10.18632/oncotarget.6871

**Published:** 2016-01-09

**Authors:** Qian Zhao, Zhongtang Wang, Wei Huang, Qiang Wang, Shuzeng Yu, Tao Zhou, Dan Han, Zhenying Wu, Heyi Gong, Hongfu Sun, Jian Zhang, Yumei Wei, Hongsheng Li, Zicheng Zhang, Haiqun Lin, Baosheng Li

**Affiliations:** ^1^ Department of Radiation Oncology, Shandong Cancer Hospital and Institute, Shandong Academy of Medical Sciences, Jinan, Shandong, P.R. China; ^2^ Department of Radiation Oncology, Shandong Cancer Hospital and Institute, Jinan, Shandong, P.R. China; ^3^ Department of Radiation Oncology, People's Hospital of Linzi District, Zibo, Shandong, P.R. China; ^4^ Department of Radiation Oncology, LiaoCheng People's Hospital, LiaoCheng, Shandong, P.R. China; ^5^ Department of Radiation Oncology, Second People's Hospital of Dezhou City, Dezhou, Shandong, P.R. China

**Keywords:** chemoradiotherapy, clinical feasibility, vinorelbine, pemetrexed, non-small-cell lung cancer

## Abstract

Our aim was to evaluate the efficacy and safety of cisplatin with pemtrexed or vinorelbine and concurrent late course accelerated hyperfractionated radiotherapy (LCAHRT). Patients with unresectable stage III non-small-cell lung cancer (NSCLC) were randomly assigned to two regimens. The experimental (PP) arm included cisplatin, pemtrexed and concurrent LCAHRT based on bilateral lung V20 = 33%. The control (NP) arm used cisplatin, vinorelbine with the same radiotherapy protocol. The primary endpoint was overall survival. Median survival times were 26.0 months (95% CI 23.2 to 28.7 months) and 28.5 months (95% CI 17.1 to 39.9 months) for the NP and PP arms, respectively (*P* = 0.26). Median progression-free survival was 12.5 months and 17.5 months in the NP and PP arms (*P* = 0.07). In both arms of the study, there were no differences in overall survival between patients with squamous and nonsquamous NSCLC. The incidences of grade 3 or 4 toxicity were higher in NP than PP arm. With concurrent LCAHRT, pemetrexed/cisplatin was equally as efficacious as vinorelbine/cisplatin, but showed a more favorable toxicity profile.

## INTRODUCTION

Lung cancer is the leading cause of cancer-related deaths worldwide [1]. Only 14% to 20% of patients with stage III locally advanced lung cancer are potentially suitable for radical resection because of invasion of adjacent tissues and/or lymph node metastasis [2, 3]. For patients with unresectable stage III non-small cell lung cancer (NSCLC), the combination of systemic chemotherapy and thoracic radiotherapy (TRT) is now the established standard treatment [4].

It has been proven that concurrent chemoradiotherapy (CCRT) confers a long-term survival benefit at no additional cost over sequential delivery of chemotherapy and TRT to NSCLC patients [5-8]. Further studies to identify the optimal chemotherapy drug combinations concurrent with radiotherapy are in progress. A randomized phase III study by the West Japan Thoracic Oncology Group (WJTOG) [9], which compared third-generation chemoradiotherapy regimens at reduced doses with second-generation regimens at full doses, has suggested that third-generation regimens significantly reduced side effects but failed to prolong patient survival.

Thanks to its favorable toxicity profile, pemetrexed is a promising agent for use in CCRT. Numerous phase I studies have shown that pemetrexed administered at its full dose with CCRT is feasible and active [10, 11, 12]. The Cancer and Leukemia Group B (CALGB) conducted a phase II study of carboplatin and pemetrexed with or without cetuximab in patients with NSCLC. They reported that pemetrexed with cetuximab could be recommended as safe for use at its full systemic dose in future trials with concurrent TRT [13]. In addition, the apparent absence of severe toxicity and the high response rates when administering pemetrexed with cisplatin (DDP) and late course accelerated hyperfractionated radiotherapy (LCAHRT) in our earlier phase I study [14] was also encouraging. However, it remains to be determined which has the superior efficacy and toxicity profile for patients with stage III NSCLC: concurrent chemoradiotherapy using pemetrexed or vinorelbine. We therefore designed a multicenter, randomized, controlled, phase III clinical trial to compare the two third-generation regimens.

## RESULTS

### Patient characteristics

Between August 2008 and September 2012, 105 patients were registered in the study. Of those, three patients did not receive the protocol treatment because they were deemed ineligible for the study as a result of being stage IV (*n* = 1), based on a physician's decision (*n* = 1) or due to performance status 3 (*n* = 1). Two patients refused to participate in the trial. The trial did not complete accrual and closed in October 2012. One hundred patients were eligible for analysis (48 for the NP arm and 52 for the PP arm). Baseline patient characteristics and demographics are listed in Table 1. There were no statistically significant differences between the two arms with respect to age, performance status, sex, histological subtype and clinical stage.

**Table 1:** Patient demographics and clinical characteristics

Demographic or Clinical Characteristic	NP arm (*n* =48)	PP arm (*n* =52)	*P*
	*n* (%)	*n* (%)	
Sex			0.148
Male	40 (83)	37 (71)	
Female	8 (17)	15 (29)	
Age, years			0.099
Median	57.4	60.3	
Range	34-73	40-75	
ECOG PS			0.886
0	6 (13)	7 (13)	
1	42 (87)	45 (87)	
Histology			0.466
Squamous	32 (67)	31 (60)	
Adenocarcinoma	15 (31)	20 (38)	
NSCLC, differentiated	1 (2)	0 (0)	
Large cell	0 (0)	1 (2)	
AJCC, Stage			0.744
IIIA	20 (42)	20 (38)	
IIIB	28 (58)	32 (62)	
Prescribed TTD			0.247
Median (range) in Gy	66.9 (51.2-79.2)	68.0 (54.0-80.6)	
Consolidation			0.312
Chemotherapy	42 (88)	45 (87)	
No	6 (12)	7 (13)	

Abbreviations: n = number; ECOG = Eastern Cooperative Oncology Group; PS = Performance status; NSCLC = non-small-cell lung cancer; AJCC = American Joint Commission on Cancer staging; TTD = total tumor dose.

### Treatment administration

Of the 100 patients, 95 patients completed the CCRT, and 87 patients completed at least two cycles consolidation chemotherapy. The median total radiation dose was 67.5 ± 6.9 Gy, which equaled a median dose in 2 Gy fractions (EQD2, T, α/β = 8.67 [15]) of 65.9 ± 6.5 Gy. In the NP arm, the median delivered dose was 66.9 Gy (range, 51.2 to 79.2 Gy), with an average delivered dose of 66.2 Gy. In the PP arm, the median delivered dose was 68.0 Gy (range, 54.0 to 80.6 Gy) and the average dose was 67.1 Gy. There was no significant difference between the median radiation dose (*P* = 0.25) in the NP and PP arms.

No patient interrupted radiation because of treatment-related toxicity, although two patients declined to continue because the schedule was too exhausting. Three patients were interrupted during chemotherapy. In the NP arm, a dose reduction and a delay of treatment were given to two patients because of grade 4 hematological toxicity. In the PP arm, one patient stopped prematurely because of seriousness eczema on his penis.

### Short-term outcome

The objective response rates were 91.7% and 88.5% in the NP and PP arms, respectively (Table 2). Although the response rate in the NP arm was superior to that in the PP arm, this difference was not statistically significant (*P* = 0.34).

**Table 2:** Objective response

Response	NP arm (*n* = 48)		PP arm (*n* = 52)	
	*n*	%	*n*	%
CR	9	18.8	17	32.7
PR	35	72.9	29	55.8
SD	3	6.2	5	9.6
PD	1	2.1	1	1.9
ORR (CR, PR)	44	91.7	46	88.5
DCR (CR. PR, SD)	47	97.9	51	98.1

Abbreviations: n = number; CR = complete response; PR = partial response; SD = stable disease; PD = progressive disease; ORR = objective response rate; DCR = disease control rate.

### Survival outcome

With median a follow-up time of 29.0 months (range, 3-74 months), the median survival time (MST) was 26.0 months (95% CI, 23.2 to 28.7 months) in the NP arm and 28.5 months (95% CI, 17.1 to 39.9 months) in the PP arm (*P* = 0.26, Figure 2). Median PFS was 12.5 months (95% CI, 9.48 to 15.5 months) in the NP arm and 17.5 months (95% CI, 11.6 to 23.4 months) in the PP arm (*P* = 0.07, Figure 3). Although there was no clinically significant difference in PFS between the two groups, there was a trend in favor of the PP arm. Overall 2-, 3- and 4-year survival rates were respectively 56.2% (95% CI, 49.0% - 63.4%), 30.3% (95% CI, 23.3% - 37.3%) and 19.0 (95% CI, 12.2-25.8) in the NP arm. The corresponding values were 53.5% (95% CI, 46.5% - 60.5%), 44.7% (95% CI, 37.6% - 51.8%) and 28.1% (95% CI, 19.4%-36.8%) in the PP arm.

**Figure 1:**
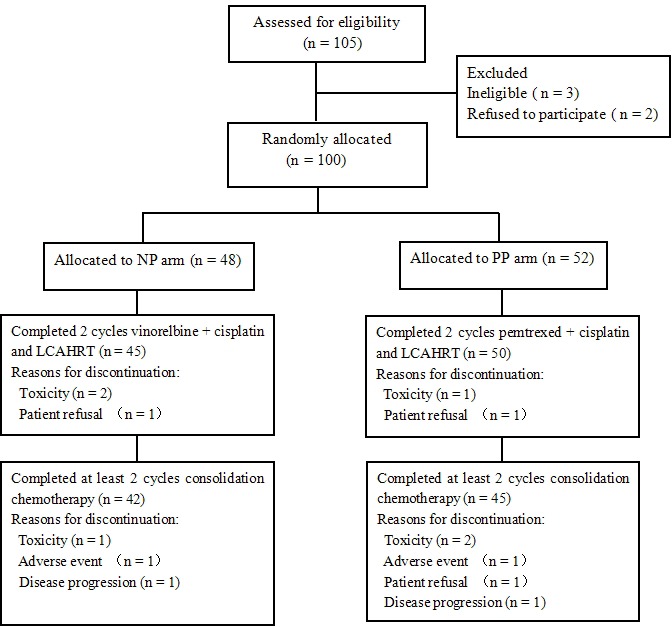
CONSORT diagram LCAHRT, late course accelerated hyperfractionated radiotherapy.

**Figure 2:**
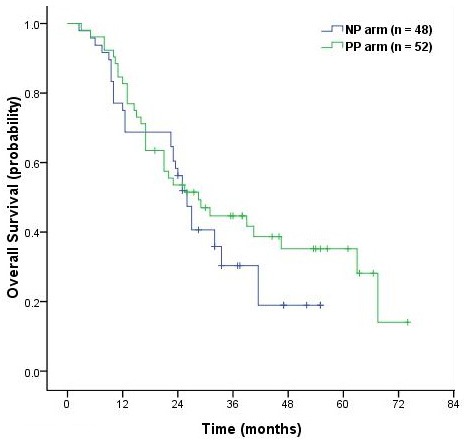
Kaplan-Meier curves of overall survival in the NP and PP arms

**Figure 3:**
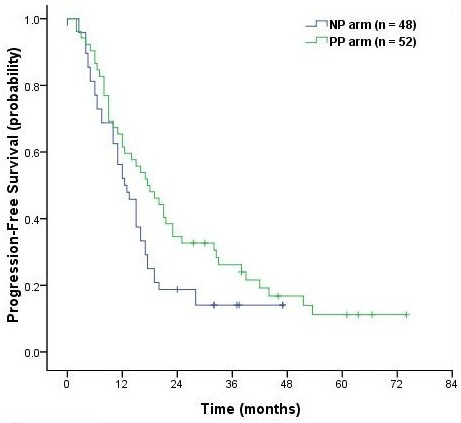
Kaplan-Meier curves of progression-free survival in the NP and PP arms

The MSTs were 25.0 and 27.0 months for the NP arm (*P* = 0.53), and 25.5 months and not reached for the PP arm (*P* = 0.16). There were no significant differences in OS between patients with squamous cell carcinoma (SCC) and non-squamous cell carcinoma (N-SCC) in the two arms. Multivariable analysis using treatment, age, KPS, sex, histology, stage, and the dose of TRT showed that, after controlling for treatment, there were no significant differences associated with survival.

### Pattern of relapse

Relapses were noted in 68 of the 100 patients. Table 3 summarizes the patterns of the sites of first relapse. There was no difference between the two arms (*P* = 0.84).

**Table 3:** Patterns of failure

Sites of Failure	NP arm (*n* =48)		PP arm (*n* = 52)	
	*n*	%	*n*	%
Locoregional recurrence only	12	25.0	11	21.1
Distant metastasis only	16	33.3	17	32.6
Locoregional + distant	5	10.4	7	13.4

Abbreviations: n = number.

### Safety

Treatment-related toxicities are summarized in Table 4. The common hematologic side effects in the NP and PP arms were leukopenia and anemia. There were significantly higher rates of leukopenia and anemia in the NP than PP arm. The rates of observed grade 3-4 leukopenia were 20/48 and 11/52 (*P* = 0.027) for the NP and PP arms, respectively, and the rates of grade 3-4 anemia were 8/48 and 5/52 (*P* = 0.047). The incidences of other adverse events, including esophagitis, nausea, vomiting and thrombocytopenia, did not significantly differ.

**Table 4:** Grade≥3 toxicities observed in the NP and PP arm

	NP arm (*n* =48)				PP arm (*n* =52)				*P*
	Grade		Grade≥3		Grade		Grade≥3		
	3	4	*n*	%	3	4	*n*	%	
Hematologic toxicity	23	8	31	64.6	13	6	19	36.5	0.090
Anemia	5	3	8	16.7	5	0	5	9.6	0.047
Leukopenia	17	3	20	41.7	7	4	11	21.2	0.027
Thrombocytopenia	1	2	3	6.3	1	2	3	5.8	0.103
Non-hematological toxicity	6	0	6	12.5	3	0	3	5.8	0.305
Nausea/vomiting	1	0	1	2.1	3	0	3	5.8	0.245
Esophagitis	3	0	3	6.3	0	0	0	0	0.149
Pulmonary fibrosis	1	0	1	2.1	0	0	0	1.9	0.732
Pneumonitis	1	0	1	2.1	0	0	0	0	0.032

In the NP arm, grades 1, 2, 3 and 4 radiation pneumonitis (RP) were respectively observed in 31 (64.5%), 8 (16.6%), 1 (2%) and 0 (0%) of the patients, while in the PP arm they were respectively observed in 21 (40.4%), 10 (19.2%), 0 (0%) and 0 (0%) of patients (*P* = 0.021). One patient in the NP arm died of pulmonary hemorrhage. The most notable non-hematological adverse event during this phase was esophagitis, which developed to grade 2 or worse in 31 patients: 19 (39.6%) in the NP arm and 12 (23.3%) in the PP arm. No severe late esophageal side effects such as severe stricture or perforation were identified.

## DISCUSSION

The results of our phase III trial show no significant difference in OS between the NP and PP arms of the study. This is the first phase III trial designed to compare the PP and NP combinations when administered with concurrent LCAHRT to patients with unresectable stage III NSCLC. From the viewpoint of toxicity, the PP regimen was superior to the NP regimen.

In the context of chemotherapy and concurrent TRT for advanced lung cancer, a number of third-generation regimens have been evaluated to identity the optimal chemotherapy regimen [16, 17]. Oh et al [18] reported similar outcomes between third-generation regimens entailing paclitaxel, docetaxel or gemcitabine and cisplatin with concurrent TRT. In addition, Wang et al [19] compared weekly paclitaxel/carboplatin and cisplatin/etoposide plus TRT, but detected no significant favorable trend toward survival with either regimen. These two studies did not show third-generation regimens with concurrent TRT to be superior with respect to survival or toxicity in NSCLC patients.

It was noteworthy that, in the present study, toxicity was significantly lower in the PP than NP arm, and there was a trend toward increased PFS in the PP arm. The published outcomes observed the recent phase III studies by Senan et al [20] are consistent with the present study. They investigated PP *versus* etoposide/cisplatin (EP) plus TRT in patients with advanced nonsquamous NSCLC and observed no significant difference in OS or PFS between the PP and EP arms. However, a significantly lower incidence of grade 3/4 adverse events was observed in the PP arm.

We used LCAHRT on the basis of a prior meta-analysis showing that the use of accelerated or hyperfractionated radiotherapy led to a significant benefit for OS [21]. Repopulation is one of the major factors that hyperfractionated and/or accelerated radiotherapy may better protect against and so improve OS [22, 23]. In the 555-patient trial conducted by Senan et al [20], MSTs were 25.0 and 26.8 months in the EP and PP arms, respectively. The median survival time of 28.5 months in the PP arm in our trial was superior to that in the treatment arm of Senan's study [20]. This survival difference might be due to the use of individualized LCAHRT based on normal tissue constraints. Another trial explored the use of NP with concurrent individualized accelerated radiotherapy based on the normal tissue dose (mean lung dose = 19 Gy) [24]. The MST (95% CI) of 25.0 (19.8-30.3) months was similar to that in the NP arm of our study.

The overall survival of SCC patients in the PP arm in this study was unexpectedly good. Our findings suggest that SCC patients treated with PP and LCAHRT experience an overall survival period similar to N-SCC patients. In the study conducted by Govindan et al [13], patients were randomly assigned to pemetrexed and CCRT with or without cetuximab. Histological analysis revealed no significant difference in OS between the SCC and N-SCC patients in the two arms. The study of Gadgeel et al [25] confirmed the same results. Because the number of patients is small in the randomized clinical trials, the statistical power for the assessment of survival is low. The use of pemetrexed-based CCRT should be studied further in a large sample of patients with NSCLC classified based on histology types.

The incidence of adverse events noted in the PP arm was significantly better than in the NP arm, particularly with respect to anemia, leukopenia and pneumonitis (*χ2* test *p* < 0.05), though the incidence of grade 3 to 4 esophagitis was not greater than previously reported [26-31]. The trial of Jenkins et al [32] confirmed that V20 was a useful factor for predicting the risk of the developing pneumonitis and can aid the selection of optimal treatment plans. The results of our study revealed that based on bilateral lung V20 = 33%, severe PR using LCAHRT appears to be well tolerated.

The limitations of this study are as follows. The study was terminated short of the planned goal because of slow accrual and a lack of improvement in survival with the pemetrexed/cisplatin treatment. Nonetheless, the incidence of grade 3 or worse toxicity in the PP arm was markedly lower than in the NP arm. Our study enrolled a substantial percentage of patients with SCC. The efficacy of pemetrexed in CCRT is superior in patients with nonsquamous histology was not known when the study was designed. The unplanned subgroup analysis showed no significant difference in survival among patients with squamous and nonsquamous histology in PP regimen. However, due to the small sample number, the study had inadequate power to draw definitive conclusions.

In conclusion, our present findings demonstrate that concurrent pemetrexed with cisplatin and LCAHRT was equally efficacious with a more favorable toxicity profile than NP. However, we will need to wait for the final results of a larger trial to confirm those benefits for the treatment of patients with locally advanced NSCLC.

## MATERIALS AND METHODS

### Patient eligibility

Patients with newly histologically diagnosed, unresectable stage III NSCLC were eligible for this study (Figure 1). Eligible patients were required to have an Eastern Cooperative Oncology Group (ECOG) performance status of 0-1, to be between 18 and 75 years old, and to have lost no more than 5% of their weight over the 3 months before enrollment. Laboratory requirements were as follows: a leukocyte count of ≥ 4,000/μL, platelets of ≥ 100,000/μL, a hemoglobin level ≥ 8 g/dL, a serum creatinine level ≤ 1.5 mg/dL, a bilirubin < 1×upper limit of normal (ULN), AST and ALT < 2.5×ULN, and alkaline phosphatase < 3×ULN. For staging, all patients underwent CT of the thorax and abdomen and either a brain CT scan or magnetic resonance imaging. A radio isotopic bone scan was also performed for all patients. Positron emission tomography was strongly recommended for enrollment. All the patients signed an informed consent form that was approved by their institutional review board prior to enrollment. Patients were randomly assigned to the two treatment arms after telephoning the trials center.

### Treatment

The vinorelbine-cisplatin (NP) regimen entailed intravenous (IV) vinorelbine 25 mg/m^2^ on days 1 and 8 and IV cisplatin 25 mg/m^2^ on days 1, 2 and 3; cycles were repeated at 21-day intervals. The pemetrexed-cisplatin (PP) regimen entailed IV pemetrexed at 500 mg/m^2^ on day 1 and cisplatin at 25 mg/m^2^ on days 1-3; cycles were repeated at 21-day intervals. In the absence of disease progression or unacceptable toxicity, patients in both arms received at least 2 cycles of consolidation chemotherapy with paclitaxel 45 mg/m^2^ or vinorelbine 25 mg/m^2^ on days 1 and 8 and cisplatin 25 mg/m^2^ on days 1, 2 and 3.

In both arms, chemotherapy dose modifications were based on the leukocyte and platelet counts. If severe hematologic toxicity occurred on a treatment day, chemotherapy was mandatorily held until recovery and then resumed at a 75% or 50% dose, as specified. If these toxicities did not abate within 6 weeks from day 1 of the previous chemotherapy cycle, subsequent cycles were stopped.

LCAHRT was administered on the first day of chemotherapy, with the RT given in two phases with no split. Irradiation was administered as conventionally fractionated radiotherapy (CFRT) entailing 5×2 Gy per week (total dose 40 Gy) in the first phase and LCAHRT in the second phase entailing 1.4 Gy twice a day, with a minimum interval of 6 hours between fractions. An individualized prescribed dose was defined based on bilateral lung V20 = 33% (the volume of the whole lung receiving > 20 Gy). The dose-volume constraints on organs at risk used for plan optimization were as following: bilateral lung, V20 = 35%; heart, V65 ≤ 33% (the volume of the whole heart receiving ≥ 65 Gy) and V45 ≤ 67% (the volume of the whole heart receiving ≥ 45 Gy); liver, V35 ≤ 50% (the volume of the whole liver receiving ≥ 35 Gy), spinal cord, < 45 Gy and esophagus, Dmax ≤ 80 Gy).

All patients were treated with intensity-modulated radiotherapy (IMRT). The CBCT scans were prospectively acquired weekly in order to register tumor volume changes. Treatment planning was based on CT simulation. The total gross tumor volume (GTV) consisted of the primary tumor and all lymph nodes greater than 1.0 cm in short axis measurement on CT, or demonstrated positive on a FDG-PET/CT scan. In particular, the target area of the primary lesion was delineated in the routine lung window, and the mediastinal target area was delineated in the mediastinal window. In patients with atelectasis, delineation of GTV was based on fused PET-CT images. Regional nodes, including the contralateral hilar, contralateral mediastinal and supraclavicular lymph nodes, were included in the GTV on the condition that imaging manifestations were positive. The clinical tumor volume (CTV) was created by adding a 5-mm margin to the GTV for squamous carcinoma and a 7-mm margin to the GTV for nonsquamous carcinoma. The planning target volume (PTV) was defined as the CTV plus an anisotropic margin of 0.5 cm for uncertainties of expectation of primary tumor movement, respiratory motion and some setup errors. The goal was to deliver the prescribed dose to at least 95% of the CTV with 95%-105% dosimetric uniformity and 90%-110% coverage of the PTV, while meeting normal tissue constraints according to the respective protocol.

### Response evaluation and toxicity

All eligible patients received a physical examination and blood chemistry studies once a week and CT scan after every two cycles of chemotherapy. The follow-up evaluations consisted of a history, physical examination, and a thoracic CT performed every 3 months during the first 2 years, and every 6 months thereafter. Other imaging examinations were obtained when recurrence was suspected. The Response Evaluation Criteria in Solid Tumors (RECIST) were used to evaluate objective response rate (ORR) as complete response (CR), partial response (PR), stable disease (SD) or progressive disease (PD).

Chemotherapy-related toxicity was judged on the basis of patients' complaints and physical examination, and was graded chiefly at the discretion of the treating physicians, according to the common terminology criteria (CTC) version 3.0.

### End-points and statistical analysis

The primary endpoint of this study was overall survival (OS). Secondary endpoints were progression-free survival (PFS) and toxic effects. OS was observed from the dates of randomization until death or last follow-up time. PFS was defined as the time from the dates of randomization to the occurrence of disease progression (whichever occurred first) or death. ORR was defined as the sum of patients with confirmed CR and PR. The Kaplan-Meier method was used to evaluate OS and PFS. The log-rank test was used to compare survival curves for different groups. Toxic effects were examined using the Pearson Chi-Square test. Values of *P* < 0.05 were considered significant, and statistical tests were based on a two-sided significance level.

It was projected that the PP regimen would improve the 2-year OS probability of 10% from the previous NP regimen. Allowing for a 10%-15% ineligibility rate, the plan was to accrue a total of 120 patients in each arm. Statistical analyses were performed using SPSS software version 17.0 (SPSS Inc., Chicago, IL).
